# The importance of N-glycosylation on β_3_ integrin ligand binding and conformational regulation

**DOI:** 10.1038/s41598-017-04844-w

**Published:** 2017-07-05

**Authors:** Xiulei Cai, Aye Myat Myat Thinn, Zhengli Wang, Hu Shan, Jieqing Zhu

**Affiliations:** 1 Blood Research Institute, BloodCenter of Wisconsin, Milwaukee, WI 53226 USA; 2 Department of Biochemistry, Medical College of Wisconsin, Milwaukee, WI 53226 USA; 3 College of Animal Science and Veterinary Medicine, Qingdao Agricultural University, Qingdao, 266109 China

## Abstract

N-glycosylations can regulate the adhesive function of integrins. Great variations in both the number and distribution of N-glycosylation sites are found in the 18 α and 8 β integrin subunits. Crystal structures of α_IIb_β_3_ and α_V_β_3_ have resolved the precise structural location of each N-glycan site, but the structural consequences of individual N-glycan site on integrin activation remain unclear. By site-directed mutagenesis and structure-guided analyses, we dissected the function of individual N-glycan sites in β_3_ integrin activation. We found that the N-glycan site, β_3_-N320 at the headpiece and leg domain interface positively regulates α_IIb_β_3_ but not α_V_β_3_ activation. The β_3_-N559 N-glycan at the β_3_-I-EGF3 and α_IIb_-calf-1 domain interface, and the β_3_-N654 N-glycan at the β_3_-β-tail and α_IIb_-calf-2 domain interface positively regulate the activation of both α_IIb_β_3_ and α_V_β_3_ integrins. In contrast, removal of the β_3_-N371 N-glycan near the β_3_ hybrid and I-EGF3 interface, or the β_3_-N452 N-glycan at the I-EGF1 domain rendered β_3_ integrin more active than the wild type. We identified one unique N-glycan at the βI domain of β_1_ subunit that negatively regulates α_5_β_1_ activation. Our study suggests that the bulky N-glycans influence the large-scale conformational rearrangement by potentially stabilizing or destabilizing the domain interfaces of integrin.

## Introduction

Glycosylation not only adds extra molecular mass to a protein and helps maintain protein stability, folding, and solubility, but also contributes to another level of structural and functional diversity^1, 2^. The attachment of carbohydrate moieties to the amide nitrogen of asparagine (Asn, N) residue, a process named N-linked glycosylation, is one of the most abundant post-translational modifications of protein^2, 3^. It has been widely appreciated that protein N-glycans play important roles in many cellular processes such as cell adhesion and migration by modulating the function of cell adhesion molecules including integrins^4–10^. Aberrant N-glycosylations have been observed under pathological conditions such as inflammation and cancer progression and metastasis^4, 11–17^, underscoring the importance of understanding the molecular function of N-glycans.

**Figure 1:**
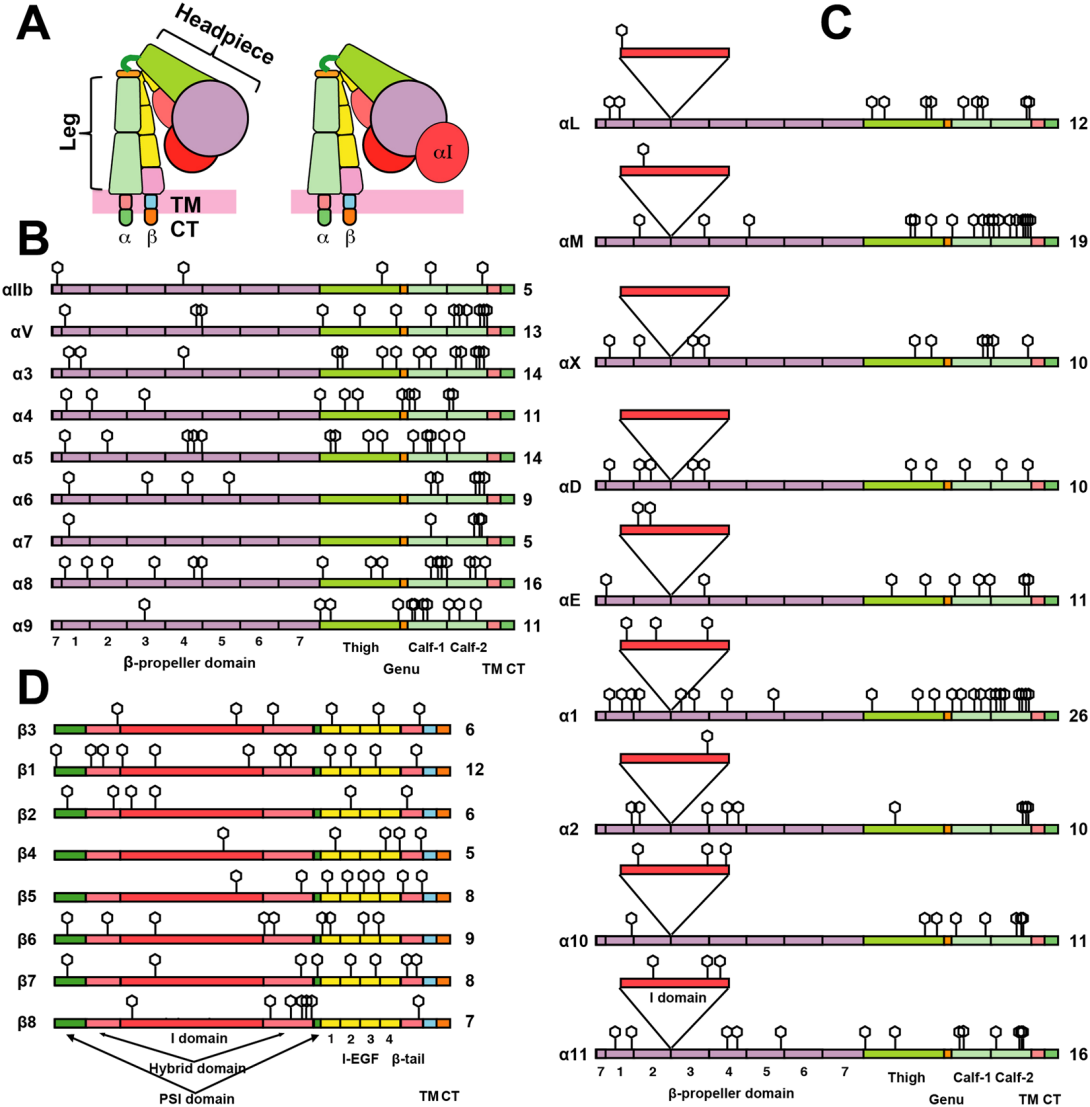
Integrin structure and N-linked glycosylation. **(A)** Cartoon models of integrin in the bent conformation. The domains are color-coded as same as panels **B**–**D**. **(B**,**C)** The distribution of potential N-glycan sites in the integrin α subunits without (**B**) or with (**C**) the αI-domain. The 7 blades of β-propeller domain are labeled. **(D)** The distribution of potential N-glycan sites in the integrin β subunits. The numbers of predicted N-linked glycosylation sites are shown on the right for each integrin subunit.

Integrins are α/β heterodimeric cell surface glycoproteins that mediate a wide range of biological functions such as development, immune response, and blood clotting^18^. The combination of 18 α and 8 β subunits results in 24 integrin members in human^18^ (Fig. 1). Each subunit of integrin contains a large extracellular domain with multiple subdomains, a single transmembrane domain and generally a short cytoplasmic domain. The integrin extracellular domain can be divided into the headpiece and the leg domains (Fig. 1A). Recent structural and functional studies have revealed that integrins can undergo a transition from a bent conformation in the resting state to an extended conformation in the active state as a result of the headpiece extension, headpiece opening and leg domain separation^19^. Such long-range conformational rearrangements are critical for the upregulation of integrin affinity to bind the extracellular ligands^19^. Both α and β integrin subunits are the major carriers of N-glycans (Fig. 1). The importance of integrin N-glycans has been evidenced by the functional effects on integrin expression, cell adhesion, spreading and migration upon the loss or gain of N-glycan sites or the changes in N-glycan contents^8, 14, 20–23^. Given the large-scale conformational changes of integrin and the bulky N-glycans attached to the moving domains of integrin, it is tempting to speculate that the N-glycans might influence the structural changes and thus the activation of integrins. In line with this possibility, a recent study on EGF receptor (EGFR) demonstrated that the N-glycosylation is critical for the ectodomain conformational rearrangement and its orientation relative to the cell membrane^24^. However, how the individual N-glycan regulates integrin conformation and ligand binding has not been well studied.

Among the integrin family, the α_IIb_β_3_ and α_V_β_3_ integrins have been very well characterized both structurally and functionally^25–30^. α_IIb_β_3_ is essential for platelet-mediated hemostasis and thrombosis^31, 32^, while α_V_β_3_ is important in tumor angiogenesis, metastasis, and inflammation^33, 34^. It has been reported that the α_V_β_3_ glycosylation differs significantly between primary and metastatic melanoma cells^14^. Although the glycan structures are shaved and only partially resolved in the crystal structures of α_IIb_β_3_ and α_V_β_3_, the precise location of each individual N-glycan site can be readily defined. Many of these N-glycan sites lie in the domain interfaces that will be rearranged or disrupted from the bent to the extended conformational transition during integrin activation. The goal of this study is to investigate the effect of loss of individual N-glycan sites on β_3_ integrin activation and relate the function of the N-glycan sites to their structure location. We also extended our study to α_5_β_1_ integrin and identified one N-glycan site of β_1_ subunit that negatively regulates α_5_β_1_ ligand binding.

## Results

### Distribution of the N-linked glycans on integrins

The potential N-linked glycosylation sites of integrins were predicted based on the presence of the consensus NXT/S sequons (X is any amino acids except proline). As shown in Fig. 1, the putative N-linked glycosylation sites are distributed among almost all the extracellular subdomains of both α and β subunits within the headpiece and leg domains (Fig. 1). Of the 18 human α integrins, half of them contain an extra ligand-binding αI (inserted) domain (Fig. 1A–C). The numbers of N-linked glycosylation sites range from 5 (α_IIb_ and α_7_) to 16 (α_8_) among the αI-less α-subunits (Fig. 1B), and from 10 (α_2_, α_D_ and α_X_) to 26 (α_1_) among the αI-containing α-subunits (Fig. 1C). The β-subunits have relatively less N-glycan sites compared with the α-subunits, ranging from 5 (β_4_) to 12 (β_1_) sites (Fig. 1D). The locations, as well as the numbers of N-glycan sites, vary substantially even within the same subdomains among both α and β subunits. Interestingly, the N-glycan sites are mostly abundant in the leg domains of both α and β subunits, especially in the calf-1 and calf-2 domains of α-subunits (Fig. 1B,C). The α_M_ and α_1_ subunits have the most abundant N-glycans (12 sites) in their calf-1 and -2 domains (Fig.1C). Some of the N-glycan sites, such as the ones adjacent to the transmembrane domains of both α and β subunits are relatively conserved (Fig. 1B–D).

### Effect of the loss of individual N-glycan sites of α_IIb_ subunit on α_IIb_β_3_ integrin expression and ligand binding

**Figure 2:**
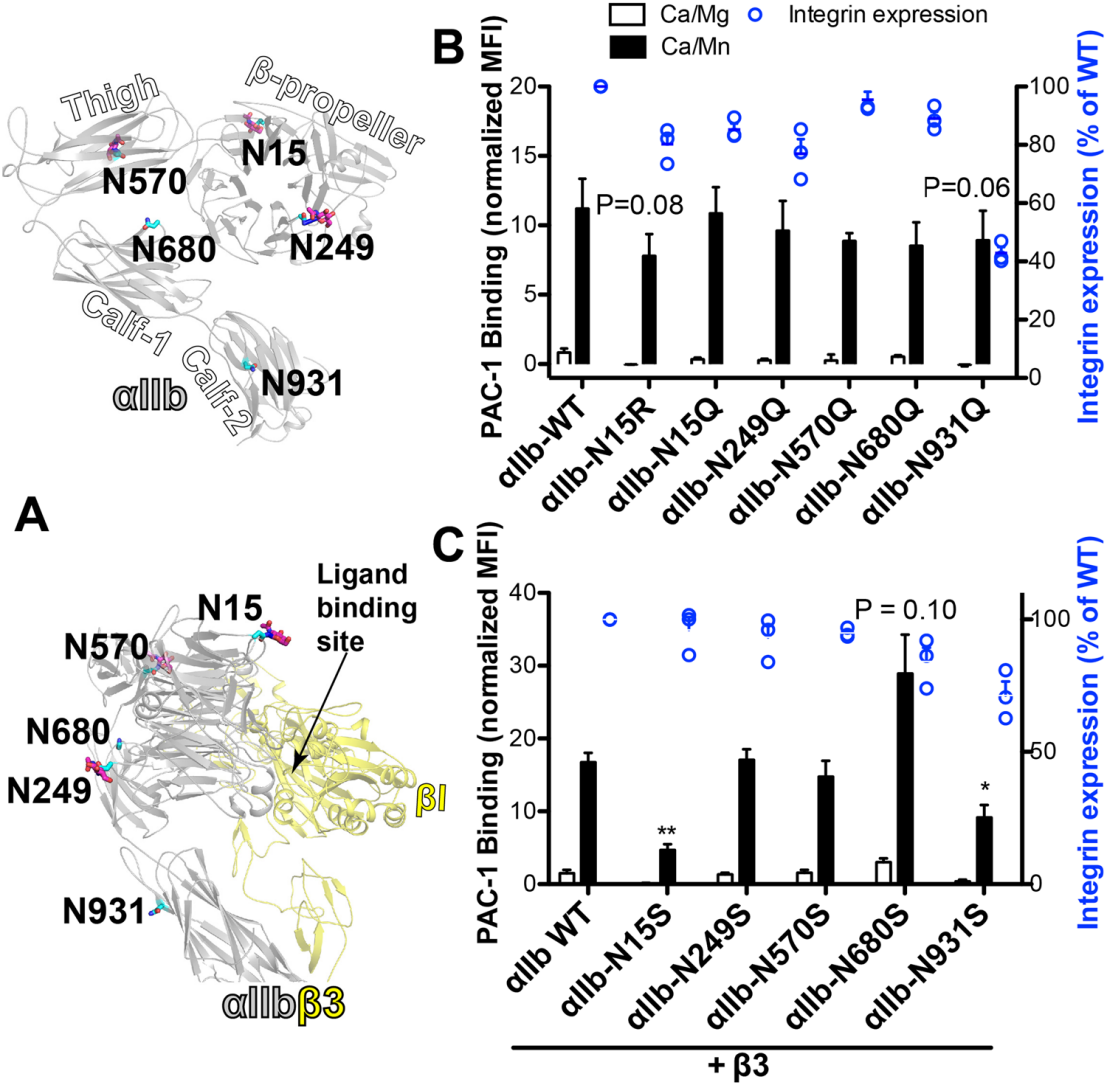
Effect of the individual N-glycan deletion of α_IIb_ subunit on α_IIb_β_3_ ligand binding. **(A)** Locations of α_IIb_ N-glycan sites in the crystal structure of α_IIb_β_3_ (PDB code 3FCS) at the bent conformation. Asn residues are shown as sticks with carbons in cyan. N-glycan residues resolved in the crystal structure are shown as sticks with carbons in magenta. Oxygens and nitrogens are red and blue, respectively. It should be noted that the N-glycan residues visualized in the crystal structure are trimmed ones. The native N-glycan chains could be much longer and more complex. **(B**,**C)** Ligand-mimetic mAb PAC-1 binding of the indicated single mutations of α_IIb_ co-expressed with β_3_ in HEK293FT cells in the presence of 1 mM Ca^2+^/ Mg^2+^ (Ca/Mg) or 0.2 mM Ca^2+^ plus 2 mM Mn^2+^ (Ca/Mn). PAC-1 binding was measured by flow cytometry and presented as mean fluorescence intensity (MFI) normalized to integrin expression (AP3 binding). Data are means ± s.e.m. (n ≥ 3). Two-tailed t-tests were used to compare the wild type (WT) and the mutants in the Ca/Mn condition. *P < 0.05; **P < 0.01.

α_IIb_β_3_ integrin has been used as a prototype in understanding integrin structure and function^32^. The α_IIb_ subunit has 5 predicted N-glycan sites: two in the β-propeller domain, one in the thigh domain, one in the calf-1 domain and one in the calf-2 domain (Fig 2A). Three of them have been visualized in the crystal structure (Fig. 2A). We removed the individual N-glycan site of α_IIb_ subunit one by one by the glutamine substitution. Each Asn to Gln mutant of α_IIb_ was co-expressed with wild-type β_3_ subunit in HEK293FT cells. The α_IIb_β_3_ integrin activation was measured by the binding of ligand-mimetic mAb PAC-1 in the physiological Ca^2+^/Mg^2+^ condition or in the external integrin activator, Mn^2+^. Overall, the individual Asn to Gln substitution of α_IIb_ subunit had no significant effect on PAC-1 binding to α_IIb_β_3_ when activated by Mn^2+^ (Fig. 2B). The α_IIb_-N931Q mutation slightly reduced PAC-1 binding (Fig. 2B). Compared with the wild-type α_IIb_ subunit, the cell surface expression of α_IIb_β_3_ was decreased up to 20% among the α_IIb_ N15Q, N249Q, N570Q and N680Q mutations. However, the α_IIb_-N931Q mutation dramatically decreased the cell surface expression to more than 50% (Fig. 2B). We also performed the Asn to Ser mutation, given most of the α_IIb_ N-glycan sites locate at a loop region and the serine residue is potentially modified by O-linked glycans. Interestingly, when co-expressed with wild-type β_3_ in HEK293FT cells, the α_IIb_-N15S and the α_IIb_-N931S mutations significantly reduced Mn^2+^-induced PAC-1 binding (Fig. 2C). There is an increase of PAC-1 binding with the α_IIb_-N680S mutation, although it is not statistically significant (Fig. 2C). The α_IIb_-N249S and α_IIb_-N570S mutations had no effect on PAC-1 binding (Fig. 2C). The Ser substitutions, especially the α_IIb_-N931S mutation, had less effect on the α_IIb_β_3_ cell surface expression than the Gln substitutions (Fig. 2B,C). Overall, individual deletion of the α_IIb_ N-glycans had little or no effect on the Mn^2+^-induced ligand binding of α_IIb_β_3_ integrin. Of note, the decreased ligand binding by the α_IIb_-N15S mutation is due to the Ser substitution not the loss of N-glycan since the α_IIb_-N15Q and α_IIb_-N15R mutation had no such obvious effect (Fig. 2B).

### Effect of the loss of each individual N-glycan site of β_3_ subunit on α_IIb_β_3_ integrin expression and ligand binding

**Figure 3:**
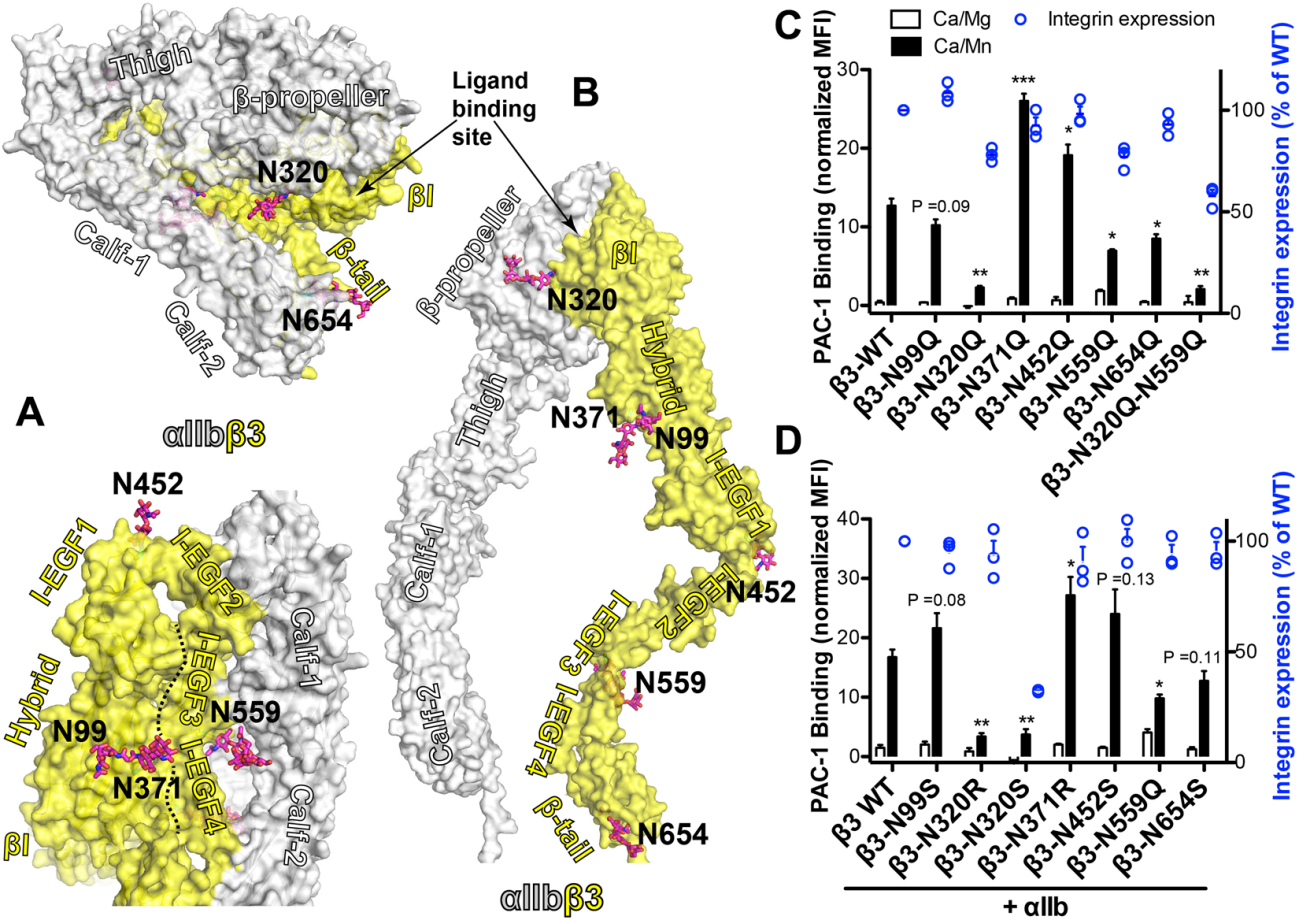
Effect of the individual N-glycan deletion of β_3_ subunit on α_IIb_β_3_ ligand binding. **(A)** Locations of the β_3_ N-glycan sites in the crystal structure of α_IIb_β_3_ (PDB code 3FCS) at the bent conformation shown as the solvent accessible surface in two views. The boundary of the hybrid/I-EGF3-4 domain interface is indicated as a black dotted line. **(B)** Model of the high affinity extended conformation of α_IIb_β_3_. Asn residues are shown as sticks with carbons in cyan. N-glycan groups resolved in the crystal structure are shown as sticks with carbons, oxygens, and nitrogens in magenta, red, and blue, respectively. **(C**,**D)** Ligand-mimetic mAb PAC-1 binding of the indicated single mutations of β_3_ co-expressed with α_IIb_ in HEK293FT cells in the presence of 1 mM Ca^2+^/Mg^2+^ (Ca/Mg) or 0.2 mM Ca^2+^ plus 2 mM Mn^2+^ (Ca/Mn). PAC-1 binding was measured by flow cytometry and presented as mean fluorescence intensity (MFI) normalized to integrin expression (AP3 binding). Data are means ± s.e.m. (n ≥ 3). Two-tailed t-tests were used to compare the wild type (WT) and the mutants in the Ca/Mn condition. *P < 0.05; **P < 0.01; ***P < 0.001.

The β_3_ subunit has 6 N-linked glycan sites: one in the βI domain, two in the hybrid domain, one in the I-EGF1 domain, one in the I-EGF3 domain and one in the β-tail domain (Fig. 3A), all of which have been resolved in the crystal structure (Fig. 3A). All these N-glycans can move with their attached subdomains during the extension of β_3_ integrin (Fig. 3B). Similar to the α_IIb_ Asn to Gln mutations, most of the β_3_ Asn to Gln single mutations had little effect on the cell surface expression of α_IIb_β_3_ in HEK293FT cells (Fig. 3C). Remarkably, the β_3_ N320Q, N559Q and N654Q mutations, located at the βI, I-EGF3, and β-tail domains, respectively, all significantly reduced the Mn^2+^-induced PAC-1 binding (Fig. 3A–C). In contrast, both the N371Q and N452Q mutations, located at the hybrid and I-EGF1 domains, respectively, significantly enhanced the Mn^2+^-induced PAC-1 binding (Fig. 3A–C). The β_3_-N320Q mutation in the βI domain had the most dramatic negative effect on PAC-1 binding among all the mutations. However, the combined mutation β_3_-N320Q-N559Q did not further decrease PAC-1 binding, but only further reduced the cell surface expression of α_IIb_β_3_ (Fig. 3C).

To test whether the mutational effect on the α_IIb_β_3_ PAC-1 binding is specific to the glutamine substitution, we also mutated the Asn to either Ser or Arg. As seen with the β_3_-N320Q mutation, both the β_3_ N320R and N320S mutations remarkably reduced the Mn^2+^-induced PAC-1 binding, although the β_3_-N320S mutation also dramatically reduced the cell surface expression of α_IIb_β_3_ (Fig. 3D). In addition, the β_3_ N371R and N452S mutations also increased PAC-1 binding, while the β_3_-N654S mutation decreased PAC-1 binding (Fig. 3D). The β_3_ N99Q and N99S mutations had no significant effect on Mn^2+^-induced α_IIb_β_3_ PAC-1 binding (Fig. 3C,D).

**Figure 4:**
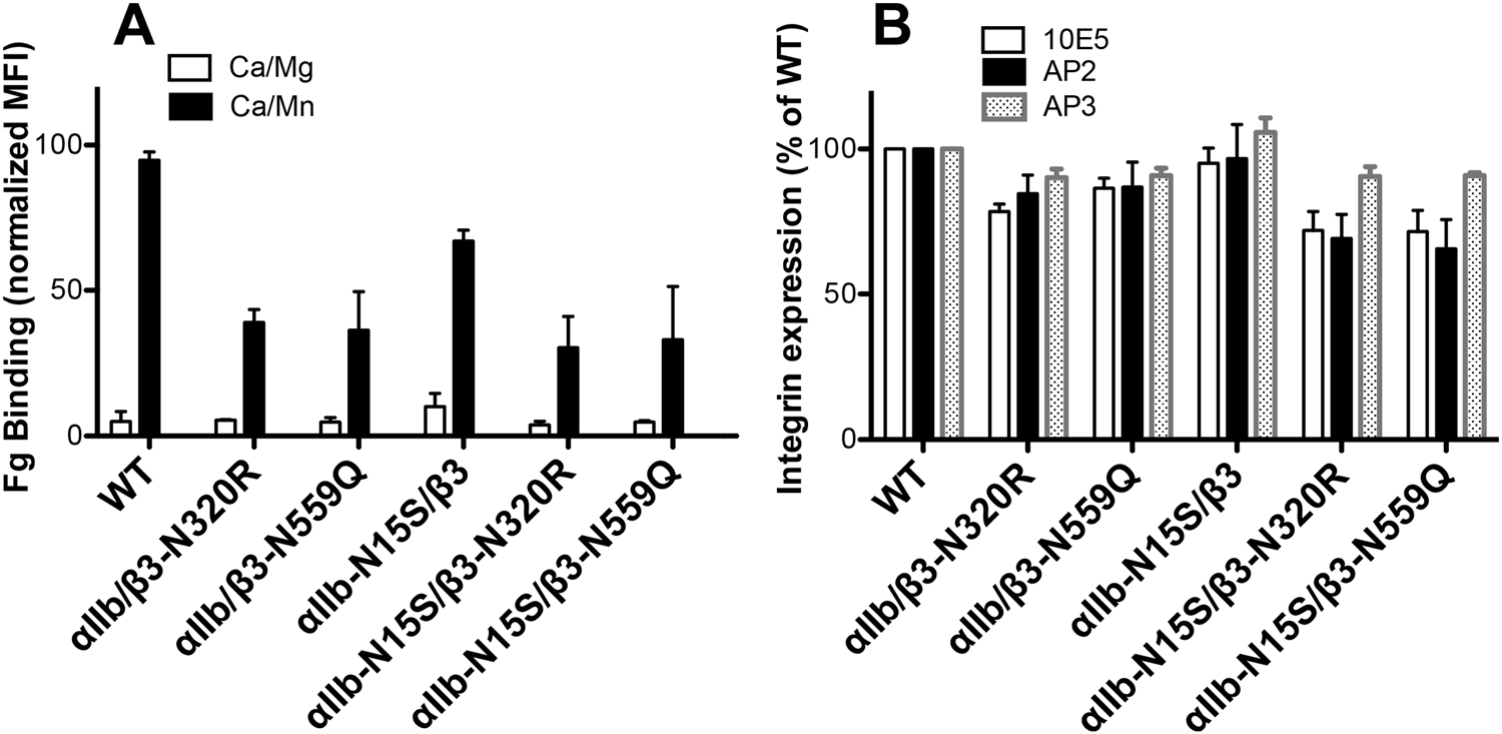
N-glycan deletions decreased α_IIb_β_3_ integrin activation from outside the cell. (**A)** Fibrinogen (Fg) binding to HEK293FT cells transfected with the indicated α_IIb_β_3_ constructs. Ligand binding was done in the presence of 1 mM Ca^2+^/Mg^2+^ (Ca/Mg) or 0.2 mM Ca^2+^ plus 2 mM Mn^2+^ (Ca/Mn). **(B)** Cell surface expression of α_IIb_β_3_ integrin constructs reported by anti-α_IIb_ mAb 10E5, anti-α_IIb_β_3_ complex-specific mAb AP2, and anti-β_3_ mAb AP3. The ligand or mAb binding was measured by flow cytometry. Data are means ± s.e.m. (n ≥ 3).

We next tested the effect of selected N-glycan mutations of α_IIb_ and β_3_ subunits on the binding of the physiological ligand human fibrinogen (Fg). Consistent with the PAC-1 binding assay, the β_3_-N320R, β_3_-N559Q and α_IIb_-N15S all decreased the Mn^2+^-induced Fg binding to α_IIb_β_3_ expressed in HEK293FT cells (Fig. 4A). However, the combined mutations, α_IIb_-N15S/β_3_-N320R and α_IIb_-N15S/β_3_-N559Q did not further reduce Fg binding (Fig. 4A). When measured by the anti-α_IIb_ mAb 10E5, the anti-α_IIb_β_3_ complex-specific mAb AP2, and the anti-β_3_ mAb AP3, these N-glycan mutations showed little effect on the cell surface expression of α_IIb_β_3_ integrin (Fig. 4B), suggesting that these N-glycans may not affect the α_IIb_ and β_3_ heterodimerization. Taken together, these data demonstrate the importance of individual N-glycan sites in regulating α_IIb_β_3_ ligand binding.

### Effect of the loss of individual N-glycan sites on α_IIb_β_3_ activation from inside the cell

**Figure 5:**
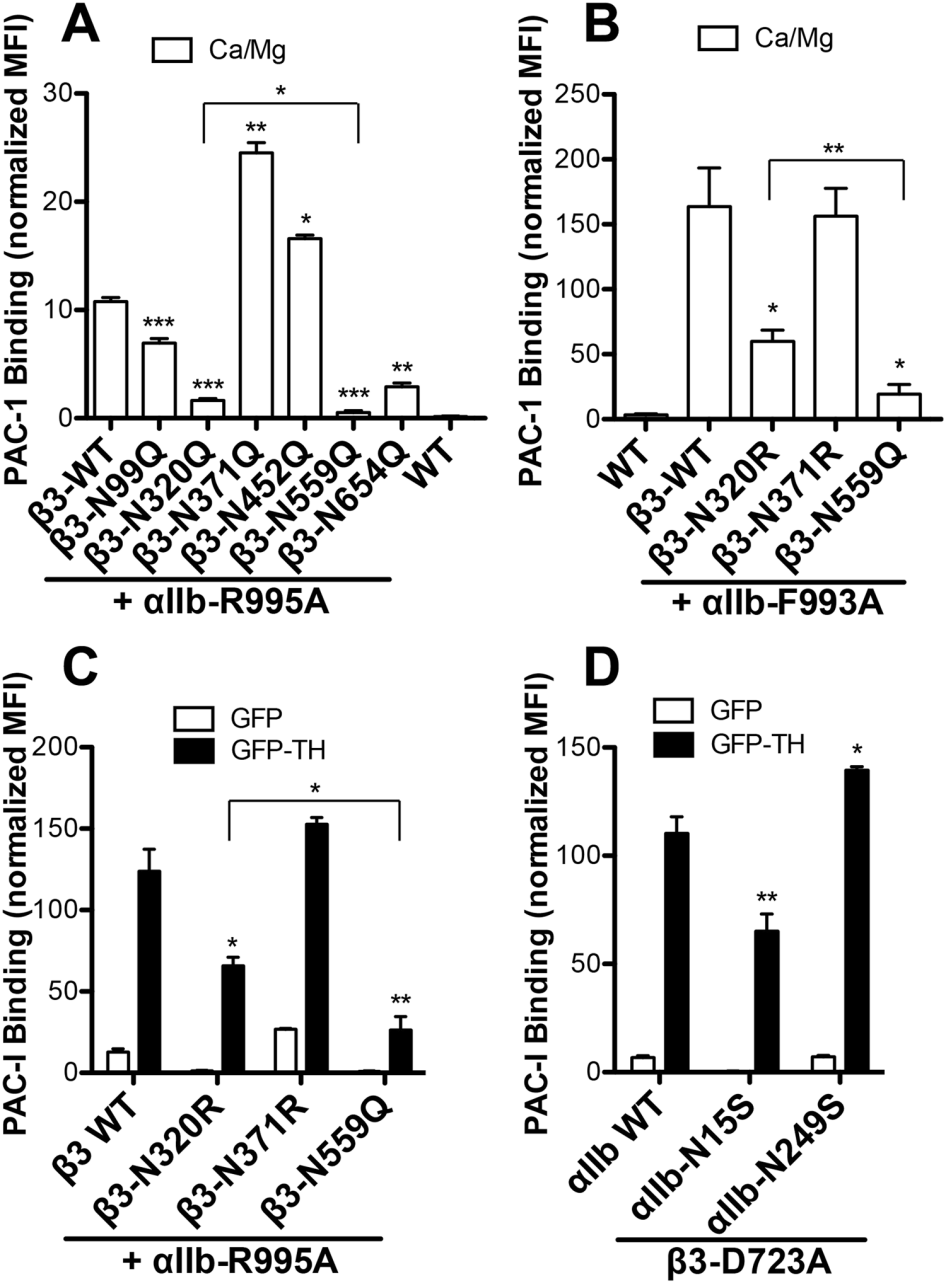
N-glycan deletions decreased α_IIb_β_3_ integrin activation from inside the cell. (**A**,**B)** PAC-1 binding of HEK293FT cells transfected with the indicated β_3_ constructs and the α_IIb_-R995A or α_IIb_-F993A mutant. **(C**,**D)** PAC-1 binding of the α_IIb_β_3_ constructs co-expressed with EGFP or EGFP-Talin1-head (TH) in the HEK293FT cells. The binding was done in the presence of 1 mM Ca^2+^/Mg^2+^ (Ca/Mg) and measured by flow cytometry. Data are means ± s.e.m. (n ≥ 3). Two-tailed t-tests were used to compare the wild type (WT) and the mutants in the same condition. *P < 0.05; **P < 0.01; ***P < 0.001.

Integrin activation can be triggered from both outside and inside the cell, namely outside-in and inside-out signaling^35^. Having found that the loss of individual N-glycan sites can exert either negative or positive effect on α_IIb_β_3_ activation induced by Mn^2+^ from outside the cell, we asked whether it has the similar effect on integrin inside-out activation, in which the signals are initiated from the cytoplasmic domain and transmitted to the ligand-binding site through large-scale conformational changes. The active mutations in the cytoplasmic domains such as the α_IIb_-R995A and α_IIb_-F993A or the overexpression of talin-1 head (TH) domain have been used to mimic integrin inside-out activation^36–38^. When co-expressed with the active α_IIb_-R995A mutation, the β_3_ N99Q, N320Q, N559Q and N654Q mutations all significantly reduced the constitutive PAC-1 binding to α_IIb_β_3_ (Fig. 5A), while the β_3_ N371Q and N452Q mutations significantly enhanced PAC-1 binding (Fig. 5A). The α_IIb_-F993A mutation rendered α_IIb_β_3_ more active than the α_IIb_-R995A mutation did (Fig. 5A,B). Likewise, the β_3_ N320R and N559Q mutations significantly reduced α_IIb_-F993A-mediated α_IIb_β_3_ activation (Fig. 5B). The β_3_-N371R mutation did not further enhance the α_IIb_-F993A-mediated PAC-1 binding probably because the PAC-1 binding already reached the maximal level (Fig. 5B). Interestingly, the β_3_-N559Q mutation in the I-EGF3 domain exerted the most profound defect on α_IIb_β_3_ inside-out activation (Fig. 5A,B).

We next tested the effect of the loss of single N-glycan sites on TH-induced α_IIb_β_3_ activation. We performed this assay in the presence of α_IIb_-R995A or β_3_-D723A mutation, which has been shown to greatly enhance the TH-induced α_IIb_β_3_ activation^39^. Consistent with the data above, the β_3_ N320R and N559Q mutations significantly reduced, while the β_3_ N371R mutation slightly increased TH-induced PAC-1 binding (Fig. 5C). As seen above, the β_3_-N559Q had a more remarkable effect than the β_3_-N320R mutation on TH-mediated α_IIb_β_3_ activation (Fig. 5C). These data demonstrate that the N-glycans can exert both negative and positive effect on α_IIb_β_3_ inside-out activation. Consistent with the Mn^2+^-induced PAC-1 binding, the α_IIb_-N15S mutation reduced TH-mediated α_IIb_β_3_ activation (Fig. 5D). In contrast, the α_IIb_-N249S mutation increased TH-mediated α_IIb_β_3_ activation (Fig. 5D). However, it should be noted again that the negative effect of α_IIb_-N15S mutation might not be directly due to the loss of N-glycan.

### Effect of N-glycan deletions on α_IIb_β_3_ integrin conformational change

**Figure 6:**
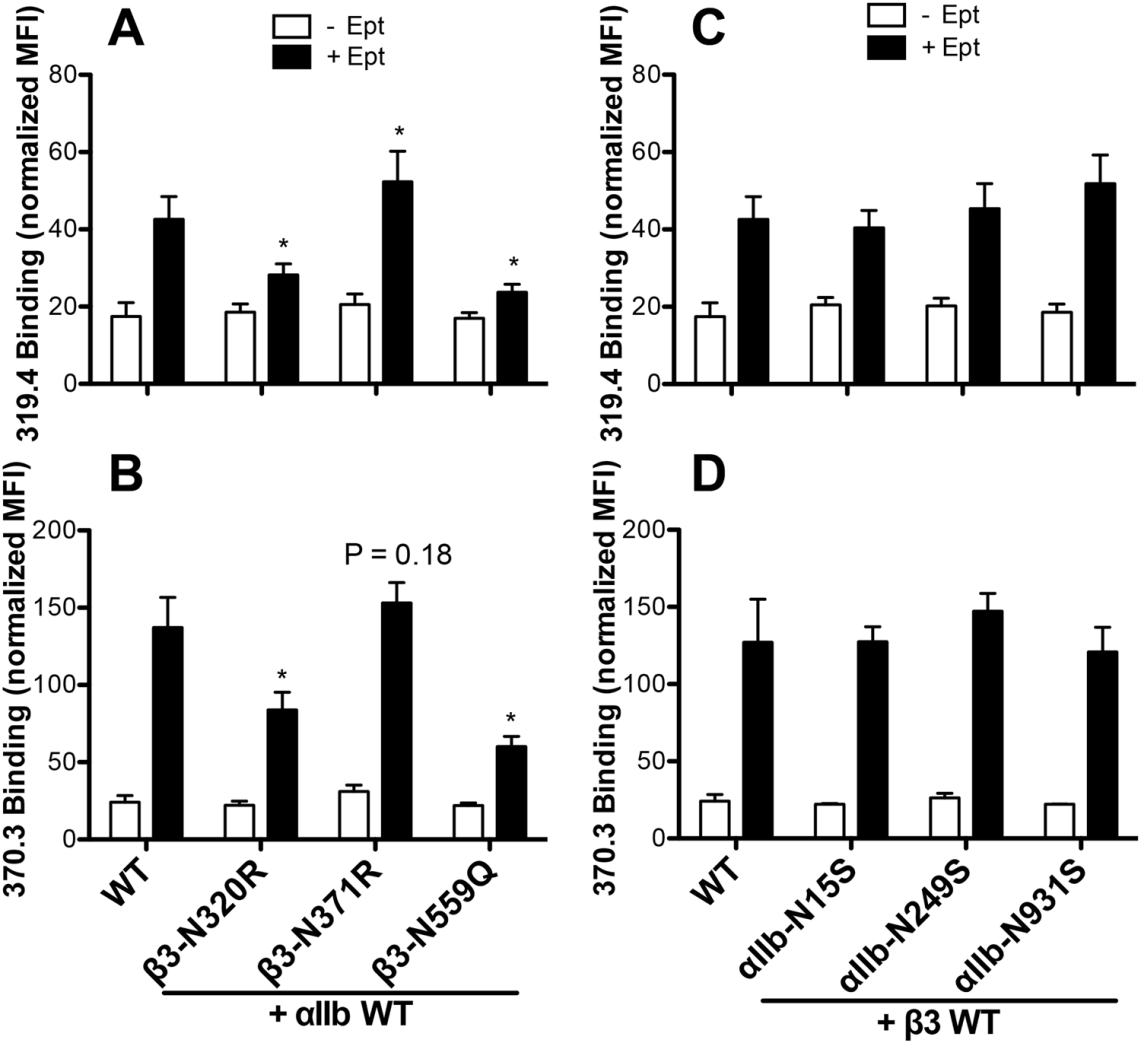
Effect of N-glycan deletions on ligand-induced conformational change of α_IIb_β_3_ integrin. (**A**–**D)** The binding of the active conformation-specific anti-β_3_ mAb 319.4 (**A**,**C**) and the anti-α_IIb_ mAb 370.3 (**B**,**D**) to the indicated α_IIb_β_3_ constructs expressed in HEK293FT cells in the absence or presence of ligand-mimetic drug eptifibatide (Ept) plus 1 mM Ca^2+^/Mg^2+^. The mAb binding was measured by flow cytometry and presented as a normalized MFI to integrin expression (AP3 binding). Data are means ± s.e.m. (n = 3). Two-tailed t-tests were used to compare the wild type (WT) and the mutants in the same condition. *P < 0.05.

The integrin affinity for ligand binding is tuned by the long-range conformational changes during integrin activation^19^. The ligand binding to the headpiece induces integrin ectodomain extension from the outside-in direction. On the other hand, activators from inside the cell also induce integrin conformational rearrangement, resulting in the affinity increase for the extracellular ligands. Since our data show that some of the N-glycans affect ligand binding of α_IIb_β_3_ integrin, which requires integrin conformational changes, it is tempting to speculate that the N-linked glycans might exert their effect through regulating the conformations of integrin. To test this possibility, we used two conformation-specific mAbs, 319.4 and 370.3, to report the active conformations of β_3_ and α_IIb_, respectively. Eptifibatide (Ept), a high-affinity ligand-mimetic inhibitor that binds both the resting and active α_IIb_β_3_, was used as a ligand to induce integrin conformational change from outside. Ept induced the binding of both 319.4 and 370.3 mAbs to α_IIb_β_3_ (Fig. 6). The β_3_ N320R and N559Q mutations significantly reduced the Ept-induced binding of both mAbs to α_IIb_β_3_ (Fig. 6A,B), while the β_3_-N371R mutation significantly enhanced the Ept-induced mAb 319.4 but not 370.3 binding (Fig. 6A,B). However, there was no obvious effect of the α_IIb_ N15S, N249S and N931S mutations on the Ept-induced mAb binding (Fig. 6C,D), although the N15S and N931S mutations reduced the Mn^2+^-mediated PAC-1 binding (Fig. 2C). These data indicate that individual N-glycans can affect the ligand-induced conformational rearrangement of α_IIb_β_3_ integrin.

**Figure 7:**
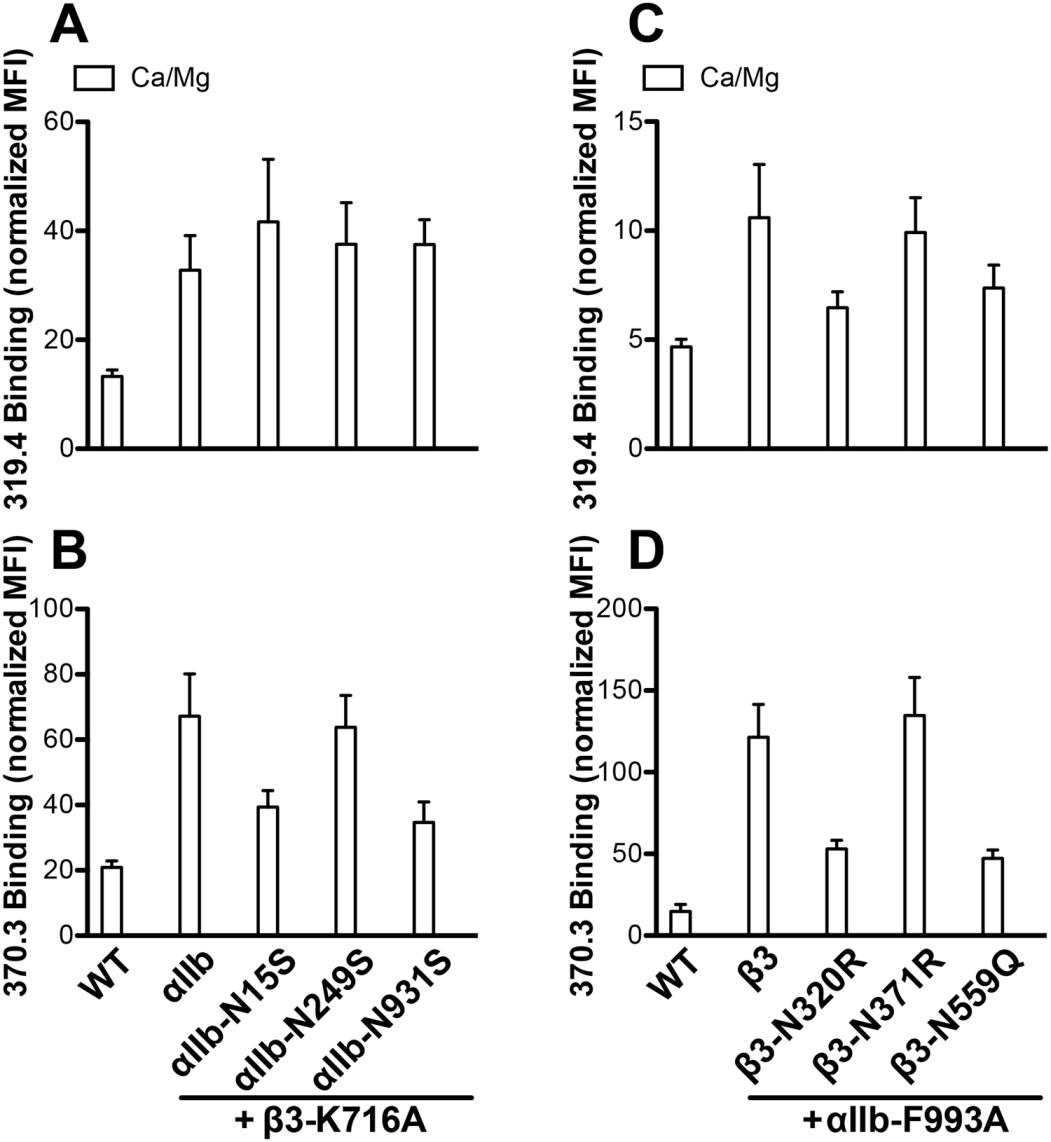
Effect of N-glycan deletions on the α_IIb_β_3_ conformational change induced by inside-out activation. (**A**,**B)** Binding of the anti-β_3_ mAb 319.4 or anti-α_IIb_ mAb 370.3 to the HEK293FT cells transfected with the α_IIb_ WT or the glycan mutants and the active β_3_-K716A mutant, which mimics integrin inside-out activation. **(C**,**D)** Binding of the anti-β_3_ mAb 319.4 or anti-α_IIb_ mAb 370.3 to the HEK293FT cells transfected with the β_3_ WT or the glycan mutants and the active α_IIb_-F993A mutant, which mimics integrin inside-out activation. Data are means ± s.e.m. (n = 3).

To test the effect of N-glycans on the conformational change of α_IIb_β_3_ integrin upon inside-out activation, we used the active cytoplasmic mutations β_3_-K716A^40, 41^ and α_IIb_-R993A. These mutations constitutively induced the binding of mAbs 319.4 and 370.3 to α_IIb_β_3_ (Fig. 7), indicating the conformational changes of integrin from the inside-out direction. The presence of α_IIb_ N15S, N249S and N931S mutations did not affect the β_3_-K716A-mediated binding of anti-β_3_ mAb 319.4 (Fig. 7A). However, the α_IIb_ N15S and N931S but not N249S mutations reduced the β_3_-K716A-mediated binding of anti-α_IIb_ mAb 370.3 (Fig. 7B). In contrast, the β_3_ N320R and N559Q mutations reduced the α_IIb_-R993A-mediated binding of both 319.4 (Fig. 7C) and 370.3 (Fig. 7D) mAbs. The β_3_-N371R mutation had no obvious effect on the α_IIb_-R993A-mediated binding of both mAbs (Fig. 7C,D) probably because the α_IIb_-R993A mutation already induced the maximum level of α_IIb_β_3_ activation as shown in the PAC-1 binding assay (Fig. 5B). These data demonstrate the importance of N-glycans in integrin conformational rearrangement during inside-out activation.

### Effect of N-glycan deletions on the activation of α_V_β_3_ integrin

**Figure 8:**
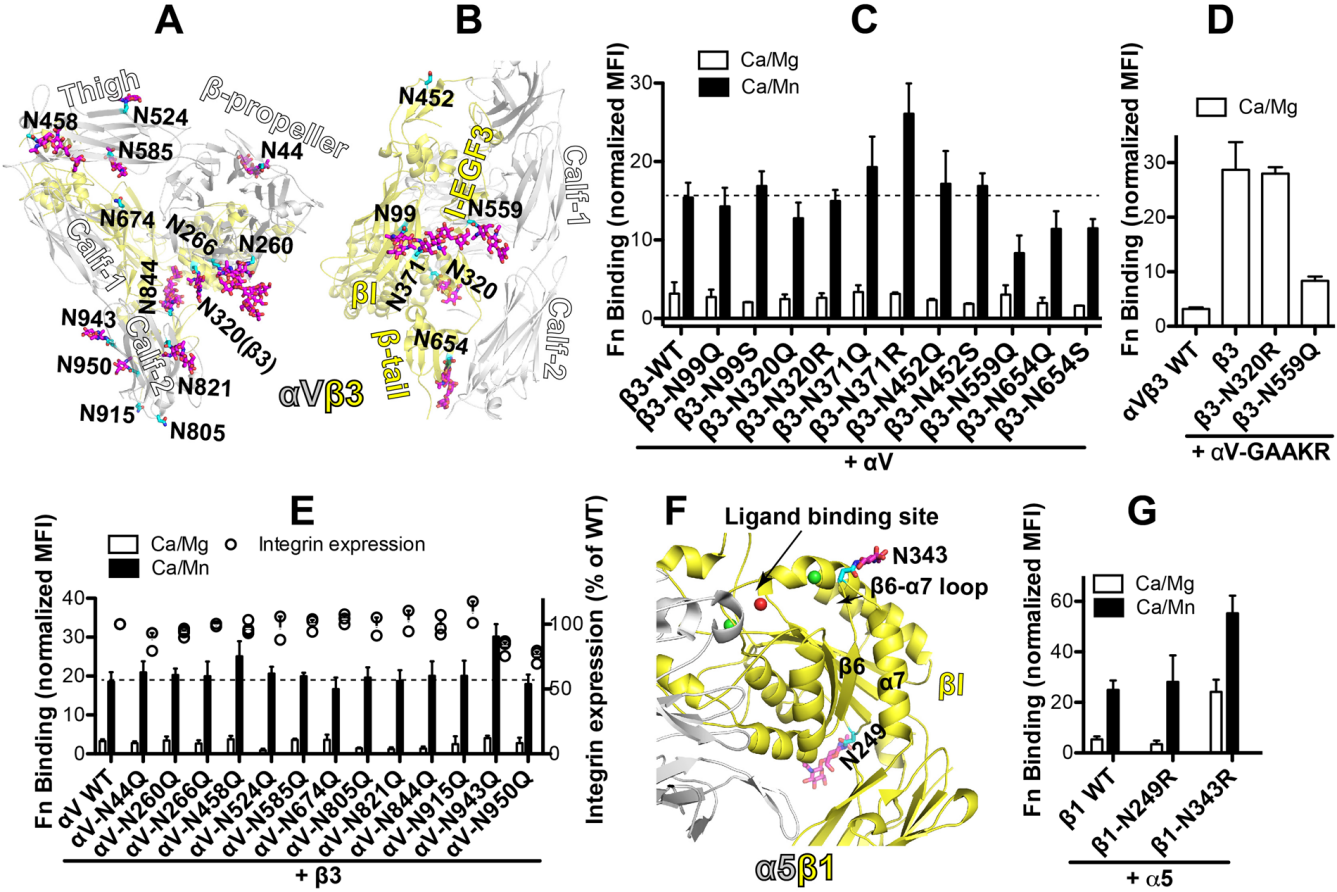
Effect of N-glycan deletions on α_V_β_3_ and α_5_β_1_ ligand binding. **(A)** Locations of α_V_ N-glycan sites in the crystal structure of α_V_β_3_ (PDB code 4G1E). **(B)** Locations of β_3_ N-glycan sites in the crystal structure of α_V_β_3_ (PDB code 4G1E). Asn residues are shown as sticks with carbons in cyan. N-glycan residues resolved in the crystal structure are shown as sticks with carbons in magenta. Oxygens and nitrogens are red and blue, respectively. **(C)** Fibronectin (Fn) binding of HEK293FT-α_5_β_1_-KO cells transfected with the β_3_ WT or the glycan mutants and α_V_ WT. **(D)** Fn binding of HEK293FT-α_5_β_1_-KO cells transfected with the indicated β_3_ constructs and the α_V_-GAAKR mutant that mimics integrin inside-out activation. **(E)** Fn binding of HEK293FT-α_5_β_1_-KO cells transfected with the α_V_ WT or the glycan mutants and β_3_ WT. **(F)** Locations of selected N-glycans at the βI domain of β_1_ integrin in the crystal structure of α_5_β_1_ headpiece (PDB code 4WJK). Asn and glycans are shown as sticks. Color codes are the same as panels A and B. Metal ions at the ligand-binding site are shown as spheres. **(G)** Fn binding of HEK293FT-α_5_β_1_-KO cells transfected with the β_1_ WT or the selected glycan mutants and α_5_ WT. Fn binding was done in the presence of 1 mM Ca^2+^/Mg^2+^ (Ca/Mg) or 0.2 mM Ca^2+^ plus 2 mM Mn^2+^ (Ca/Mn). Data are means ± s.e.m. (n = 3).

The β_3_ subunit also forms a heterodimer with α_V_ subunit. Changes in the complexity of N-linked glycosylation have been observed in α_V_β_3_ integrins during the metastatic progression of tumor cells^14^. Having established the functional role of the individual N-glycan sites in α_IIb_β_3_ integrin activation, we further studied the effect of N-glycan deletions on the function of α_V_β_3_ integrin. The attachments of N-glycans have been confirmed in the α_V_β_3_ crystal structure for most of the N-glycan sites (Fig. 8A,B). Human fibronectin (Fn) was used as a physiological ligand for α_V_β_3_. To avoid the effect from the α_5_β_1_ integrin, which is the major Fn receptor, we used the HEK293FT cells with both endogenous α_5_ and β_1_ subunits being knocked out by the CRISPR/Cas9 technology. Consistent with the α_IIb_β_3_ integrin, the β_3_-N559Q and the β_3_-N654Q/S mutation reduced, while the β_3_-N371Q/R mutation increased Mn^2+^-mediated Fn binding to α_V_β_3_ integrin (Fig. 8C). However, the β_3_-N99Q/S, β_3_-N452Q/S, and even the β_3_-N320Q/R mutation had no obvious effect on α_V_β_3_ Fn binding (Fig. 8C). When co-expressed with the activating α_V_-GAAKR mutation, which mimics α_V_β_3_ inside-out activation^42^, the β_3_-N559Q but not β_3_-N320R mutation remarkably dampened Fn binding (Fig. 8D). This is also in contrast with the α_IIb_β_3_ integrin, in which both β_3_-N559Q and β_3_-N320R mutations greatly reduced the inside-out activation of α_IIb_β_3_ (Fig. 5B). These data indicate that certain individual N-glycans of β_3_ subunit may exert different effects on the function of α_IIb_β_3_ and α_V_β_3_ integrins.

Next, we studied the effect of removal of each individual N-glycan site in α_V_ subunit on the activation of α_V_β_3_ integrin. α_V_ subunit has 13 potential N-glycan sites, 10 of which have been confirmed in the crystal structure (Fig. 8A). As shown in Fig. 8E, compared with the wild-type α_V_β_3_, most of the individual N-glycan deletions by the N to Q substitutions had no obvious effect either on the cell surface expression or the Fn binding of α_V_β_3_ (Fig. 8E). Among all the mutations, only the α_V_-N458Q and the α_V_-N943Q moderately increased Mn^2+^-induced Fn binding to α_V_β_3_ (Fig. 8E), and only the α_V_-N943Q and the α_V_-N950Q mutations mildly reduced the cell surface expression of α_V_β_3_ (Fig. 8E). We also did the Ser substitutions for each N-glycan site of α_V_ subunit, but no obvious effect was observed on Mn^2+^-induced α_V_β_3_ Fn binding (Data not shown).

### One N-glycan site in the βI domain of β_1_ subunit negatively regulates α_5_β_1_ integrin activation

Among the integrin β subunits, β_1_ subunit has the most abundant putative N-glycan sites (Fig. 1D). When paired with α_5_ subunit, the α_5_β_1_ heterodimer has 26 potential N-glycan sites. The function of N-glycans in the α_5_β_1_ complex formation and cell surface expression or in α_5_β_1_-mediated cell adhesion, migration, and interaction with other cell surface receptors has been studied using mutagenesis approach^8^. By comparing the locations of the putative N-glycan sites among the integrin β subunit (Fig. 1D), we found that the β_1_ subunit has an N-glycan site of β_1_-N343 uniquely residing at the β6-α7 loop of βI domain, which has been determined in the α_5_β_1_ headpiece crystal structure (Fig. 8F). A hallmark structural change of the βI domain during integrin activation is the downward movement of the β6-α7 loop and the α7-helix^27, 30^. Therefore, we hypothesized that the unique N-glycan of β_1_-N343 at the β6-α7 loop might play a role in regulating α_5_β_1_ activation. When co-expressed with the wild-type α_5_ subunit in the HEK293FT-α_5_β_1_-KO cells, the β_1_-N343R mutation greatly enhanced the Fn binding to α_5_β_1_ both in Ca/Mg and Ca/Mn conditions (Fig. 8G). As a control, the β_1_-N249R mutation distal to the ligand-binding site (Fig. 8F) had no effect on α_5_β_1_ Fn binding (Fig. 8G). Thus, the loss of the N-glycan at the β_1_ β6-α7 loop facilitates α_5_β_1_ integrin activation, indicating a negative regulation by this unique N-glycan of β_1_ subunit.

## Discussion

The great variation in the number and distribution of N-linked glycosylation sites among integrin subunits add another level of heterogeneity to the very complicated integrin family (Fig. 1). An increasing body of evidence indicates that integrin N-glycans contribute to cell adhesion and migration probably by affecting integrin expression, internalization, and association with other cell surface molecules^5, 15, 16^. However, a direct connection between integrin N-glycans and activation-dependent ligand binding has been missing. In addition, previous work studied the functional effect of either the overall changes in integrin glycosylation or the combined N-glycan sites such as within the same integrin subdomains^20, 43, 44^, but the function of each individual N-glycan site has not been well documented. In this study, using the structurally well-characterized α_IIb_β_3_, α_V_β_3,_ and α_5_β_1_ as model integrins, we found that the loss of certain individual N-glycan site either reduced or enhanced integrin activation reported by the changes in the binding of ligands or active conformation-specific mAbs, indicating that the N-linked glycosylation can exert both positive and negative effects on integrin function.

Among the N-glycan mutations of the α_IIb_ subunit, only the α_IIb_-N15S mutation largely reduced the Mn^2+^- and TH-induced α_IIb_β_3_ activation. It seems that the negative effect on integrin activation is not due to the loss of N-glycan of α_IIb_-N15 since the α_IIb_-N15R and α_IIb_-N15Q mutations didn’t have much effect on α_IIb_β_3_ ligand binding. A previous study showed that the α_IIb_-N15Q mutation inhibited pro-α_IIb_ maturation, complex formation, and degradation^45^, but we didn’t see an obvious effect on the cell surface expression of α_IIb_β_3_ with this mutation. The α_IIb_-N15 resides at the blade 7 of β-propeller domain close to the interface formed by the α_IIb_ β-propeller and the β_3_ βI domain. It is not readily known why the α_IIb_-N15S mutation affects α_IIb_β_3_ ligand binding. A similar N-glycan site of α_V_ integrin, α_V_-N44, locates at the blade 1 of α_V_ β-propeller domain, but its mutation to Gln or Ser had no effect on Mn^2+^-induced α_V_β_3_ Fn binding. Thus, the negative effect of α_IIb_-N15S on α_IIb_β_3_ ligand binding should be specific to the Ser residue, probably due to the gain of O-linked glycosylation as indicated by the change of molecular weight of α_IIb_-subunit (data not shown), which may affect ligand binding directly or indirectly through affecting α_IIb_β_3_ conformational change. The N-glycans and O-glycans differ in the composition and the formation of the branches within the glycan structures, which determine the interaction with other molecules^12^. Moreover, the N-glycans also vary significantly in length and complexity^12^. Although the complete deletion of α_IIb_-N15 N-glycan had no effect on α_IIb_β_3_ ligand binding, the negative effect of α_IIb_-N15S mutation by the potential gain-of-O-glycan suggests that the structure variations of α_IIb_-N15 N-glycan may regulate the α_IIb_β_3_ function, which is clearly worth further investigation.

Remarkably, almost all the β_3_ single N-glycan deletion mutations affected the α_IIb_β_3_ ligand binding induced either by Mn^2+^ or by the activating mutations that mimic integrin inside-out activation. Our structural analysis revealed an interesting pattern of the N-glycan location and its impact on integrin ligand binding. All the three β_3_ N-glycans, including N320, N559, and N654, which positively regulate α_IIb_β_3_ activation, locate at the α_IIb_-β_3_ inter-chain interfaces. The β_3_-N320 glycan locates near the interface of α_IIb_ β-propeller and β_3_ βI domain and inserts into the interface between the headpiece and the leg domains in the bent conformation of α_IIb_β_3_ (Fig. 3A). Similarly, the β_3_-N559 glycan lodges in the interface of α_IIb_ calf-1/2 and β_3_ I-EGF3 domains. The β_3_-N654 glycan resides at the interface of α_IIb_ calf-2 and β_3_ β-tail domains (Fig. 3A). All of these interfaces are disrupted during integrin activation due to the headpiece extension and leg domain separation (Fig. 3B)^19^. The hydrophilic and bulky glycan groups may destabilize these interfaces at the bent conformation by repulsive interactions and therefore facilitate integrin conformational change. Consequently, deletion of these wedge-like glycans dampens α_IIb_β_3_ ligand binding and conformational change potentially due to the stabilization of the bent inactive conformation of integrin. This is consistent with the previous studies showing that introducing an artificial N-glycan site into the βI and hybrid domain interface of β_3_ subunit^46^ or into the thigh and calf-1 domain interface of α_IIb_ subunit^47^ renders α_IIb_β_3_ constitutively active by enforcing integrin extension. In contrast, the β_3_ N-glycans, attaching to N371 and N452, which negatively regulate α_IIb_β_3_ activation, are located close to the intra-chain interfaces of β_3_ subunit. Unlike the β_3_-N559 glycan that inserts into the domain interface, the β_3_-N371 glycan lies on the interface formed by the β_3_ hybrid and I-EGF3/4 domains (Fig. 3A), which is disrupted upon the β_3_ extension (Fig. 3B). The loss of β_3_-N371 glycan increased α_IIb_β_3_ activation, indicating that this glycan contributes to stabilizing the interface highly possibly by acting like a door bolt. In addition, the loss of the β_3_-N99 N-glycan, which is adjacent to the N-glycan of β_3_-N371 but farther away from the hybrid/I-EGF interface, slightly decreased α_IIb_β_3_ activation. The close contacts between β_3_-N371 and β_3_-N99 N-glycans may influence the conformation of the β_3_-N371 N-glycan, which in turn affects the hybrid/I-EGF interface. Upon deletion of the β_3_-N99 N-glycan, the conformation of the β_3_-N371 N-glycan may further stabilize the hybrid/I-EGF interface at the bent conformation. The β_3_-N452 glycan resides at the opposite side of the acute angle formed by the I-EGF1 and I-EGF2 domains (Fig. 3A). This angle opens to almost 180° after integrin extension (Fig. 3B), which places the β_3_-N452 glycan at the newly formed I-EGF1/2 domain interface (Fig.3B). As a result, the bulky N-glycan of β_3_-N452 may exert repulsive tension to the interface and thus destabilize the extended conformation. Indeed, the removal of β_3_-N452 N-glycan facilitated α_IIb_β_3_ activation. Thus, our data demonstrate a location-specific contribution of individual N-glycan sites in regulating integrin activation largely through affecting integrin conformational changes.

A direct link between integrin N-glycans and the conformational regulation has been missing until a recent study, in which the effect of N-glycans on the conformational equilibria of α_5_β_1_ integrin was elegantly investigated^48^. Our data are consistent with their results showing that the complex N-glycans stabilize the extended active conformation relative to the bent resting conformation. It was also proposed that the bulky N-glycans might exert their regulatory roles by crowding or repulsive interactions within the domain interfaces^48^, which is in agreement with the structure interpretations of our data. However, only the combined effects of the overall changes of N-glycans on integrin affinity were examined in the α_5_β_1_ study and the results might be a mixture of both negative and positive effects, although the positive effect was obviously dominated^48^. Our data show that individual N-glycan sites exert different effects on the β_3_ integrin activation, arguing the importance of investigating the functional role of individual N-glycans in integrin activation.

Although α_V_β_3_ integrin shares the same β_3_ subunit with α_IIb_β_3_, it is influenced differently by the loss of certain β_3_ N-glycan sites. Particularly, the β_3_-N320 mutation greatly reduced α_IIb_β_3_ activation but had no effect on α_V_β_3_ activation. Moreover, the α_V_-N844 glycan, although buried at the interface between the β_3_ βI and β-tail domain in the bent conformation of α_V_β_3_ (Fig. 8A), also had no effect on Mn^2+^-mediated α_V_β_3_ Fn binding, consistent with a recent study^29^. The requirement of large-scale conformational changes for α_V_β_3_ ligand binding remains controversial^29, 49^, but an increasing body of data supports the importance of integrin extension and headpiece opening in α_V_β_3_ activation^29^. However, since the function of α_IIb_β_3_ is to mediate platelet aggregation for hemostasis, it requires the activity of α_IIb_β_3_ to be strictly regulated^31^, while the fundamental function of α_V_β_3_ is to mediate cell adhesion and migration that are essential for cell survival and proliferation^34^, and thus the activation of α_V_β_3_ might not be as strictly regulated as α_IIb_β_3_ integrin. The different effect of β_3_ N-glycans on α_V_β_3_ and α_IIb_β_3_ activation might be attributed to the differences in their biological function. Indeed, the differences in the affinity regulation between α_V_β_3_ and α_IIb_β_3_ had been reported previously^50, 51^.

The natural variants that cause the loss- or gain-of-function due to individual glycosylation mutations have not been reported for β_3_ integrins. Nevertheless, a recent study showed that aberrant glycosylation of both α_V_ and β_3_ subunits were observed between primary and metastatic melanoma cells, which modify the integrin-mediated tumor cell migration^14^. However, it is not known which N-glycan site is changed during the tumor cell progression. Moreover, N-glycans can have different types depending on their contents, including the high-mannose, hybrid, and complex N-glycans. It has been shown that the composition of N-glycans such as branching and sialylation regulates the function of N-glycans^2^. Considering the importance of individual N-glycan sites in integrin activation as determined in the present study, it will be of great interest to determine if the heterogeneity of certain individual N-glycans affects their regulatory roles in integrin function. Of the 13 N-glycan sites of α_V_ subunit, deletion of each individual site had little effect on α_V_β_3_ expression and activation. It will be interesting to know if the simultaneous removal of multiple N-glycan sites will affect integrin function as shown in the study of α_5_β_1_ integrin^20, 44^.

Among the 24 human integrins, the function of N-glycans of α_5_β_1_ has been relatively well characterized. The N-glycan sites that are important for α_5_β_1_ heterodimer formation and biological function have been determined in both α_5_ and β_1_ subunits^20, 44^. Recent studies have mapped the important biological function to several individual N-glycan sites. For example, the N-glycan sites at the α_5_ β-propeller domain are important for α_5_β_1_-mediated cell adhesion and migration^21, 52^; one of the N-glycans at the α_5_ calf-1 domain is important for the complex formation with EGFR and the inhibition of EGFR signaling^53^; the N-glycan at the β_1_ β-tail domain is important for β_1_ activation and interaction with other cell membrane proteins including syndecan-4 and EGFR^54^; the 3 N-glycan sites at the β_1_ βI domain are important for α_5_β_1_ complex formation and cell spreading^44^. Furthermore, recent kinetic studies of the correlation between α_5_β_1_ affinity and conformation suggest that the complex-type N-glycans of α_5_β_1_ help stabilize the high-affinity conformation^48^. In the current study, we identified one N-glycan site at β_1_-N343 in the βI domain, which negatively regulates β_1_ activation since the removal of this glycan site rendered α_5_β_1_ constitutively active. The β_1_-N343 glycan may directly regulate the conformational change of βI domain by restraining the movement of the β6-α7 loop, or directly regulate ligand binding due to its proximity to the ligand binding site as proposed based on the structure modeling studies^55, 56^. We expect to identify more location-specific functions of individual N-glycan sites in α_5_β_1_ and other integrins. These N-glycan sites may work in concert to balance the biological activity of integrin.

Understanding of integrin conformation and affinity regulation has been greatly assisted by the mutagenesis studies. Critical residues and interactions that are important to integrin activation have been identified based on the loss- or gain-of-function mutations^57^. By site-directed mutagenesis, our study provides evidence that individual N-linked carbohydrate residues, depending on their structural locations, regulate integrin activation at least in part through influencing the conformational rearrangement. Exactly how these N-glycan “hotspots” are modified and regulated and how they contribute to integrin affinity and biological function need to be studied in more detail using both structural and cell biology approaches.

## Materials and Methods

### DNA constructs

DNA constructs of human α_IIb_β_3_, α_V_β_3_, α_5_β_1_, and EGFP-tagged mouse talin-1-head (EGFP-TH) were as described^42, 57, 58^. All the mutations were introduced by PCR using PfuTurbo DNA polymerase following the protocol of the QuikChange XL site-directed mutagenesis kit (Agilent Technologies). The introduced mutations were confirmed by DNA sequencing (Retrogen).

### Antibodies and protein ligands

PAC-1 (BD Bioscience) is a ligand-mimetic mAb (IgM) specific for activated α_IIb_β_3_ integrin^59^. AP3 is a conformation-independent anti-β_3_ mAb^60^ and was conjugated with Alexa Fluor 488 (ThermoFisher Scientific) or Sulfo-NHS-Biotin (ThermoFisher Scientific). 10E5 is an anti-α_IIb_ mAb^27, 61^. 319.4 and 370.3 are mAbs that recognize the active conformations of β_3_ and α_IIb_, respectively^57^. Human fibrinogen (Fg) (Enzyme Research Laboratories) and human fibronectin (Fn) (Sigma-Aldrich) were conjugated with Alexa Fluor 647 (ThermoFisher Scientific). PE-labeled MAR4 (BD Bioscience) is a non-functional anti-β_1_ integrin mAb. VC5 is anti-α_5_ integrin mAb (BD Bioscience). Alexa Fluor 647 conjugated goat anti-mouse IgM and PE-conjugated streptavidin were from ThermoFisher Scientific.

### Cell lines

HEK293FT cells (ThermoFisher Scientific) were cultured in DMEM plus 10% FBS at 37 °C with 5% CO_2_. The α_5_ and β_1_ subunits double-knockout HEK293FT (HEK293FT-α_5_β_1_-KO) cells were generated by the CRISPR/Cas9 gene editing technology using the α_5_ and β_1_ CRISPR/Cas9 KO plasmids from Santa Cruz Biotechnology. In brief, cells were transfected with the α_5_ KO plasmids for 5–7 days. The α_5_-negative cells were selected by single cell sorting after staining with the anti-α_5_ mAb VC5. The established α_5_-KO cells were transfected with the β_1_ KO plasmids and the β_1_-negative cells were selected by single cell sorting after staining with the anti-β_1_ mAb MAR4. Single cell clones with the lowest expression of both α_5_ and β_1_ subunits were selected for the following experiments.

### Soluble ligand binding assay

PAC-1 and Fg binding of HEK293FT cells transfected with α_IIb_β_3_ were as described^39, 57^. For EGFP-TH-induced ligand binding, HEK293FT cells were co-transfected with integrin constructs and EGFP or EGFP-TH for at least 24 hours. Ligand binding was performed in HBSGB buffer (25 mM HEPES, pH 7.4, 150 mM NaCl, 5.5 mM glucose, and 1% BSA) with 5 μg/ml PAC-1 or 50 μg/ml Alexa Fluor 647-labeled Fg in the presence of 10 μM eptifibatide (α_IIb_β_3_-specific inhibitor) or 1 mM Ca^2+^/Mg^2+^ (Ca/Mg) or 0.2 mM Ca^2+^ plus 2 mM Mn^2+^ (Ca/Mn) at 25 °C for 30 min. Cells were then washed and incubated on ice for 30 min with the detecting reagents: 10 μg/ml Alexa Fluor 488-labeled AP3 (for Fg binding) or Alexa Fluor 488-labeled AP3 plus Alexa Fluor 647-labeled goat anti-mouse IgM (for PAC-1 binding). For EGFP-TH-induced PAC-1 binding, cells were washed and incubated on ice with biotin-labeled AP3 plus PE-labeled streptavidin and Alexa Fluor 647-labeled goat anti-mouse IgM. Human Fn binding to α_V_β_3_ or α_5_β_1_ integrins were performed with HEK293FT-α_5_β_1_-KO transfectants. Cells were first incubated with 50 μg/ml Alexa Fluor 647-labeled Fn in the presence of 10 μM cilengitide (α_V_β_3_-specific inhibitor), 5 mM EDTA (for α_5_β_1_), Ca/Mg or Ca/Mn, and then washed and incubated with 10 μg/ml Alexa Fluor 488-labeled AP3 (for α_V_β_3_) or PE-labeled MAR4 (for α_5_β_1_). Integrin positive or integrin and EGFP double-positive cells were acquired for calculating the mean fluorescence intensity (MFI) by flow cytometry using Accuri C6 cytometer (BD Biosciences). Ligand binding was presented as normalized MFI, i.e. ligand MFI (after subtracting the ligand MFI in the inhibitor or EDTA condition) as a percentage of integrin MFI. By this calculation, the binding of ligands was normalized to total integrin expression.

### Conformation-specific antibody binding

Binding of the active conformation-specific anti-β_3_ mAb 319.4 and anti-α_IIb_ mAb 370.3 to the HEK293FT transfectants was performed as described^39, 57^. In brief, cells were first incubated with 10 μg/ml biotin-labeled 319.4 or 370.3 in the HBSGB buffer containing 1 mM Ca^2+^/Mg^2+^ in the absence or presence of 10 μM α_IIb_β_3_-specific ligand-mimetic inhibitor eptifibatide at 25 °C for 30 mins, and then washed and incubated with 10 μg/ml Alexa fluor 488-labeled AP3 and Alexa fluor 647-labeled streptavidin on ice for 30 min. AP3 positive cells (expressing α_IIb_β_3_ integrin) were acquired for calculating the MFI by flow cytometry. The 319.4 or 370.3 mAb binding was presented as normalized MFI, i.e. streptavidin MFI as a percentage of AP3 MFI. By this calculation, the binding of conformation-specific mAbs was normalized to total integrin expression.

### Statistical analysis

The statistical analysis was performed using the GraphPad Prism software. Two-tailed Student’s *t*-test was used to calculate the p values for comparing the two experimental groups, for example, the wild type and the mutant data under the same condition. The assays were repeated independently at least three times for statistical analysis.

### Data Availability

The datasets generated during and/or analysed during the current study are available from the corresponding author on reasonable request.
